# pH-Controlled selection between one of three guests from a mixture using a coordination cage host

**DOI:** 10.1039/c5sc01475a

**Published:** 2015-05-07

**Authors:** William Cullen, Katie A. Thomas, Christopher A. Hunter, Michael D. Ward

**Affiliations:** a Department of Chemistry , University of Sheffield , Sheffield S3 7HF , UK . Email: m.d.ward@sheffield.ac.uk; b Department of Chemistry , University of Cambridge , Lensfield Road , Cambridge CB2 1EW , UK . Email: herchelsmith.orgchem@ch.cam.ac.uk

## Abstract

We demonstrate the use of a simple pH swing to control the selection of one of three different guests from aqueous solution by a coordination cage host. Switching between different guests is based on the fact that neutral organic guests bind strongly in the cage due to the hydrophobic effect, but for acidic or basic guests, the charged (protonated or deprotonated) forms are hydrophilic and do not bind. The guests used are adamantane-1,3-dicarboxylic acid (H_2_**A**) which binds at low pH when it is neutral but not when it is deprotonated; 1-amino-adamantane (**B**) which binds at high pH when it is neutral but not when it is protonated; and cyclononanone (**C**) whose binding is not pH dependent and is therefore the default guest at neutral pH. Thus an increase in pH can reversibly switch the host between the three different bound states cage·H_2_**A** (at low pH), cage·**C** (at medium pH), and cage·**B** (at high pH) in succession.

## Abstract 2

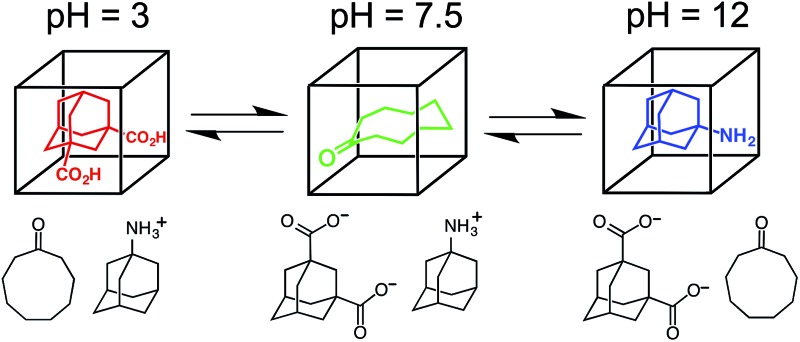
We demonstrate the use of a simple pH swing to control the selection of one of three different guests from aqueous solution by a coordination cage host.

## Introduction

The ability of self-assembled molecular containers to accommodate guest molecules in the central cavity^1^ is underpinning the development of a range of interesting functions from catalysis to drug delivery.^2^ These cavities provide a shape- and size-constricted environment that is quite different from that in bulk solution. This environment can be controlled to some extent synthetically, providing a degree of control over which types of guest can bind and under what conditions: for example, incorporation of fluorinated groups,^3^ aromatic panels^4^ or an array of H-bonding units^5^ on the internal surface of molecular containers have all been used to provide selective binding of different types of guest. Given the importance of understanding guest binding quantitatively, we^6^,^7^ and others^8^ have performed systematic studies of the specific thermodynamic contributions to guest binding in particular container families. Very recently we have shown how we could use our knowledge of guest binding properties in a specific coordination cage to develop a scoring function which allows protein/ligand docking software to predict new guest types for the cage, with high reliability, from a virtual screen:^9^ this is the first such application of the methodology of drug discovery to identifying new synthetic supramolecular host/guest systems.

Beyond the ability to design molecular containers as hosts, and to put guest binding on a quantitative and predictable footing, the next level of control is to be able to switch guest uptake/release by an external stimulus. Several examples are known of the release of guests from containers following disassembly or irreversible decomposition of the host.^10^ Reversible stimulus-responsive uptake and release of guests is much rarer, with a handful of examples including the use of a redox swing,^11^ light-induced isomerisation,^12^ or a pH swing^13^,^14^ to control guest binding. We demonstrated recently how acidic or basic guest molecules with p*K*_a_ values in the range 3–11, including some drug molecules, could undergo fully-reversible changes in binding constant of up to three orders of magnitude in a coordination cage host as the pH changed.^14^ This occurred irrespective of the sign of the charge on the guest. Thus, neutral amine guests were expelled from the cavity on protonation to give a cation, and neutral carboxylic acid guests were expelled from the cavity on deprotonation to give an anion, with the driving force in each case being the improved solvation (*i.e.* loss of hydrophobic character) in water.

After the stimulus-responsive uptake/release of individual guests, the next stage of control in host/guest complex formation would be to use the external stimulus (here, the pH swing) to switch an assembly not just between bound and unbound states, but *between several different bound states*. We describe here the first demonstration of this behavior, showing how a simple change in pH can result in one of three different guests binding in a coordination cage host, with each one being bound and then released in turn as the pH is varied. This represents a significant advance in the control that can be achieved with host/guest systems, which therefore opens the door to more sophisticated forms of functional behavior in which one of several different guests can be selected at will from a mixture.

## Results and discussion

**Fig. 1:**
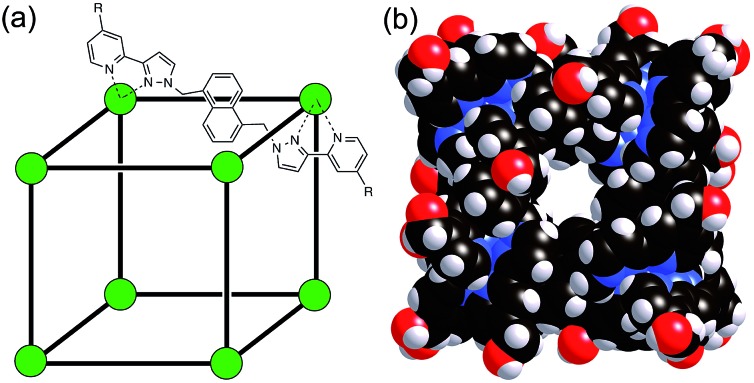
(a) Sketch of the cubic host cage showing the disposition of bridging ligands spanning each edge (R = CH_2_OH); (b) a space-filling view of the complete cage cation, showing the external O atoms of the hydroxyl groups in red (reproduced from ref. 7a).

The host cage used for these studies is the Co_8_L_12_ assembly (Fig. 1) whose host/guest chemistry we have described in previous reports.^6^,^7^,^9^,^14^ It contains a high-spin Co(ii) ion at each vertex of an approximate cube, and a bis-bidentate ligand containing two pyrazolyl-pyridine chelating termini^15^ spanning each edge of the cube. The pendant hydroxyl groups on the external surface make the cage water-soluble,^7^ and its hydrophobic interior – lined with CH groups from the ligand – results in strong binding of suitably-sized guests in water with binding constants of up to 10^8^ M^–1^.^7^,^9^,^14^ It is stable over a wide pH range, and the paramagnetism of the Co(ii) ions acts as a shift reagent dispersing the ^1^H NMR signals over the range *ca.* +100 to –100 ppm, greatly facilitating NMR-based analysis of guest binding.^6^,^7^,^14^

**Scheme 1:**
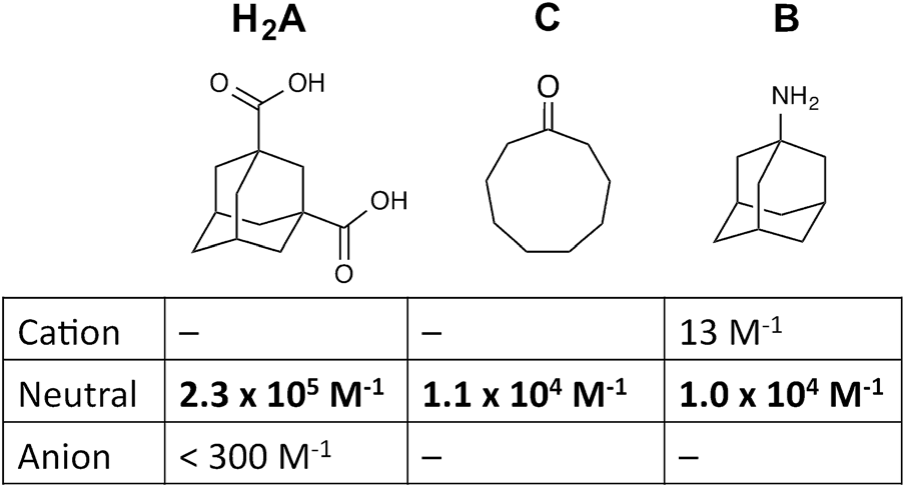
Structural formulae of the three guests used and the variation of their binding constant in the host cage with charge according to protonation/deprotonation state of the guest.

For these experiments we have selected three guests: acidic adamantane-1,3-dicarboxylic acid (H_2_**A**) which binds with *K* = 2.3 × 10^5^ M^–1^ at low pH when it is neutral, but very weakly above pH 5 when it is deprotonated to **A**^2–^;†
†.The two p*K*_a_ values for adamantane-1,3-dicarboxylic acid are 4.8 and 5.9, so we can be confident that the binding behaviour measured at pH 3 and 8 corresponds to the neutral and dianionic forms of the guest, respectively (see ref. 14).,^14^ basic 1-amino-adamantane (**B**) which binds with *K* = 1.0 × 10^4^ M^–1^ at high pH when it is neutral, but very weakly below pH 11 when it is protonated to H**B**^+^;^14^ and cyclononanone (**C**) whose binding constant of 1.1 × 10^4^ M^–1^ is pH independent.7*b* These are summarized in Scheme 1.

**Fig. 2:**
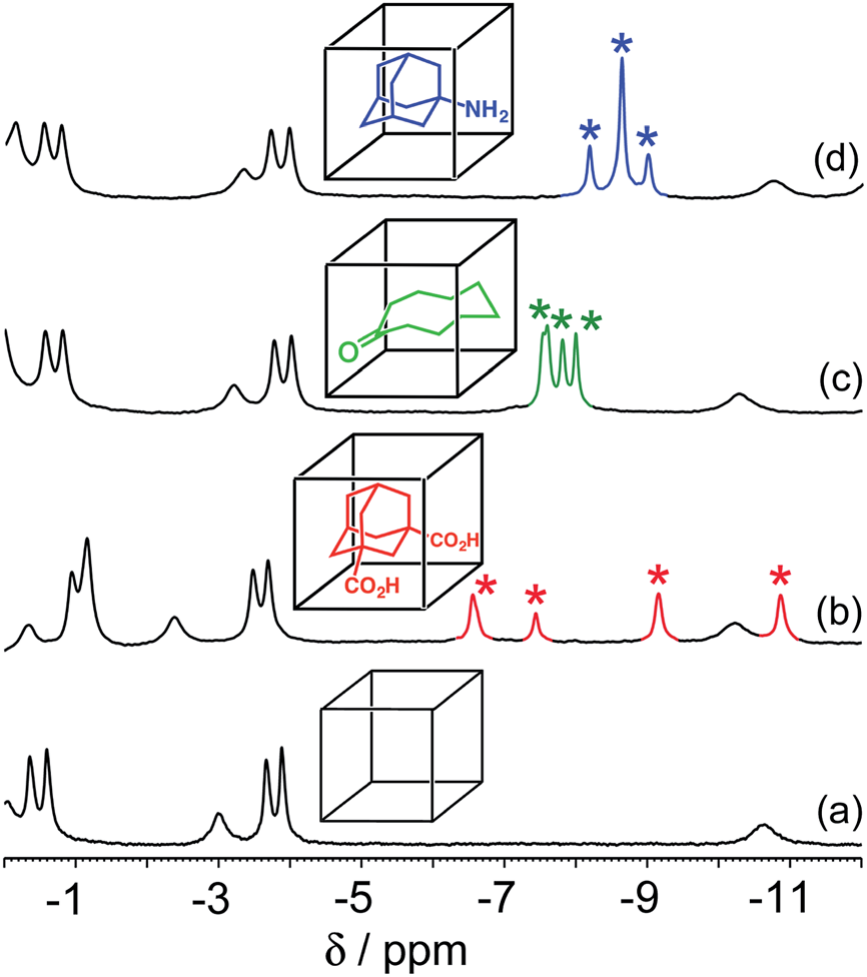
Parts of the ^1^H NMR spectra (D_2_O, 298 K) of (a) free cage; (b) complex cage·H_2_**A**; (c) complex cage·**C**; and (d) complex cage·**B**, recorded at 0.2 mM cage in the presence of excess guest such that the cage was fully bound. This region of the NMR spectrum shows signals for the bound guests (highlighted), shifted by the paramagnetism of the host cage. The different spectroscopic signatures of each bound guest are distinct and clear in the region –6 to –11 ppm.

In all cases, guest binding is signaled by a shift of the ^1^H NMR signals of the bound guest to the region –6 to –10 ppm (Fig. 2) as a consequence of the array of paramagnetic ions surrounding the bound guest in the cavity. Each guest gives a quite distinct pattern of signals in this region which, fortuitously, is clear of signals from the host cage. This provides a convenient way to monitor replacement of one guest by another as the pH changes.

Initially we performed two separate pH-based switching experiments, involving competition between guests H_2_**A** and **C**, and then between guests **B** and **C**. The protocol in every case was the same: a solution of the host cage (0.2 mM) and the two guests – at concentrations determined by their binding constant in the cage – was prepared in D_2_O and the pH was adjusted by addition of NaOD or DCl, and the ^1^H NMR spectrum and pH were recorded after each addition.‡
‡Experimental methodology was reported in detail in the ESI to ref. 14.


**Fig. 3:**
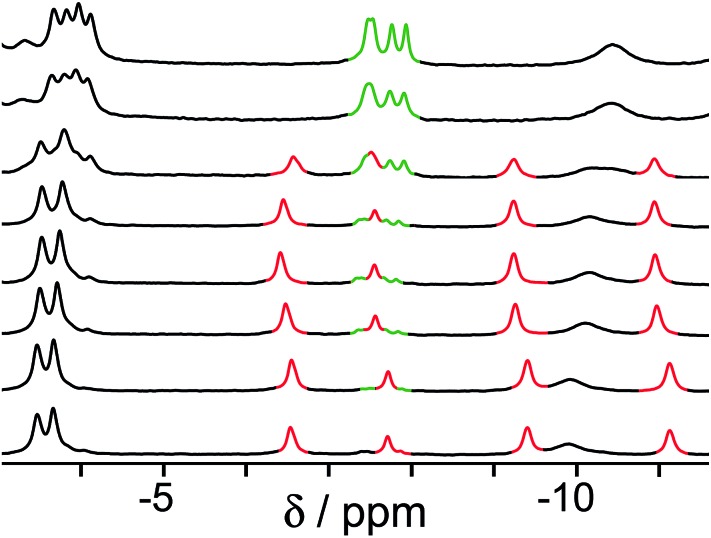
Series of ^1^H NMR spectra (D_2_O, 298 K) of a mixture of cage (0.2 mM), **C** (0.2 mM) and H_2_**A** (0.98 mM). pH values (from bottom to top): 2.0, 4.1, 4.3, 4.6, 5.0, 5.6, 7.3, 8.8. Replacement of bound H_2_**A** (red signals) by **C** (green signals) as the pH rises is clear.

**Fig. 4:**
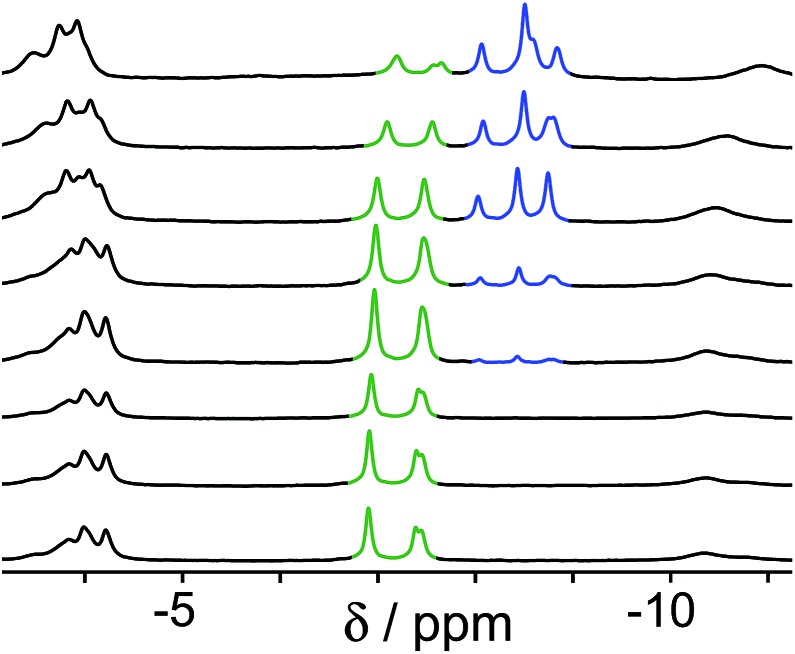
Series of ^1^H NMR spectra (D_2_O, 298 K) of a mixture of cage (0.2 mM), **C** (0.2 mM) and **B** (2.0 mM). pH values (from bottom to top): 5.0, 7.4, 8.9, 9.6, 9.8, 10.6, 11.3, 12.2. Replacement of bound **C** (green signals) by **B** (blue signals) as the pH rises is clear.

**Fig. 5:**
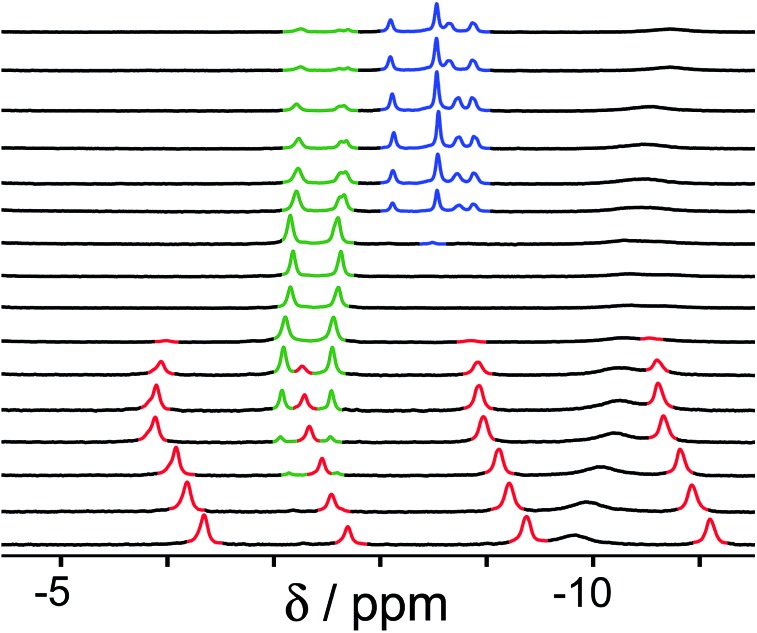
^1^H NMR spectra (D_2_O, 298 K) of a mixture of cage (0.2 mM), H_2_**A** (0.75 mM), **B** (7.1 mM) and **B** (0.2 mM), in the chemical shift region where the paramagnetically shifted signals from bound guests occur. pH values (from bottom to top): 2.0, 2.8, 4.3, 4.9, 5.7, 6.0, 6.5, 7.1, 8.1, 9.3, 9.7, 10.0, 10.6, 11.0, 11.5, 12.2. The change in occupancy of the cavity by the three different guests in succession is clear as the pH rises; the red, green and blue signals arise from the bound guests in the complexes cage·H_2_**A**, cage·**C**; and cage·**B** (see Fig. 2).

The results of the first experiment (switching between H_2_**A** and **C**) are in Fig. 3. At low pH, neutral H_2_**A** binds much more strongly than **C**, and at the concentrations used we can only detect the cage·H_2_**A** complex in the ^1^H NMR spectrum with no competing bound state cage·**C**. As the pH is raised, the characteristic signals of bound H_2_**A** decrease in intensity and are replaced by a new set of signals from bound **C** in the complex cage·**C** (Fig. 3). The physical interpretation of this is that as the pH rises and H_2_**A** is deprotonated to **A**^2–^, it becomes hydrophilic and therefore more weakly binding than **C** which is not affected by pH. Thus, H_2_**A** is replaced completely (within the limits of sensitivity of the NMR experiment – see spectrum at pH 8.8 in Fig. 3) by **C** as guest, with **A**^2–^ being ejected from the host due to its hydrophilicity.^14^ The effect is fully reversible, with binding switching between the cage·H_2_**A** and the H·**C** states as the pH is changed.§
§Note that the signals for bound cyclononanone (**C**) are slightly different in Fig. 4 and 5 compared to Fig. 2 and 3, *i.e*. their appearance is different in the presence of the non-bound **A**^2–^ in the mixture. This is consistent with some association in solution between the polycationic host cage and (free) **A**^2–^ which alters the environment of the *bound* guest slightly. We have seen this effect before, when the NMR signals of a BF_4_^–^ anion inside the cavity of a paramagnetic tetrahedral cage were sensitive to weak interactions of other anions in solution with the external surface of the cage (ref. 16).


A similar effect is seen in the experiment with guests **B** and **C** (Fig. 4). In this case the two guests have similar binding constants, so excess of **B** was used to allow binding of **B** to dominate over **C** when **B** is in its neutral form. At neutral pH, the only complex present is cage·**C**, because **B** is fully protonated as hydrophilic H**B**^+^ whose binding is very weak.^14^ As the pH rises and H**B**^+^ is deprotonated to neutral **B**, the signals for bound **C** are reduced in intensity, and a new set of signals characteristic of bound **B** grows in as cage·**C** is replaced by cage·**B**. By pH 12.2, cage·**B** is clearly the dominant complex as we would expect given the presence of excess **B** over **C**.

**Fig. 6:**
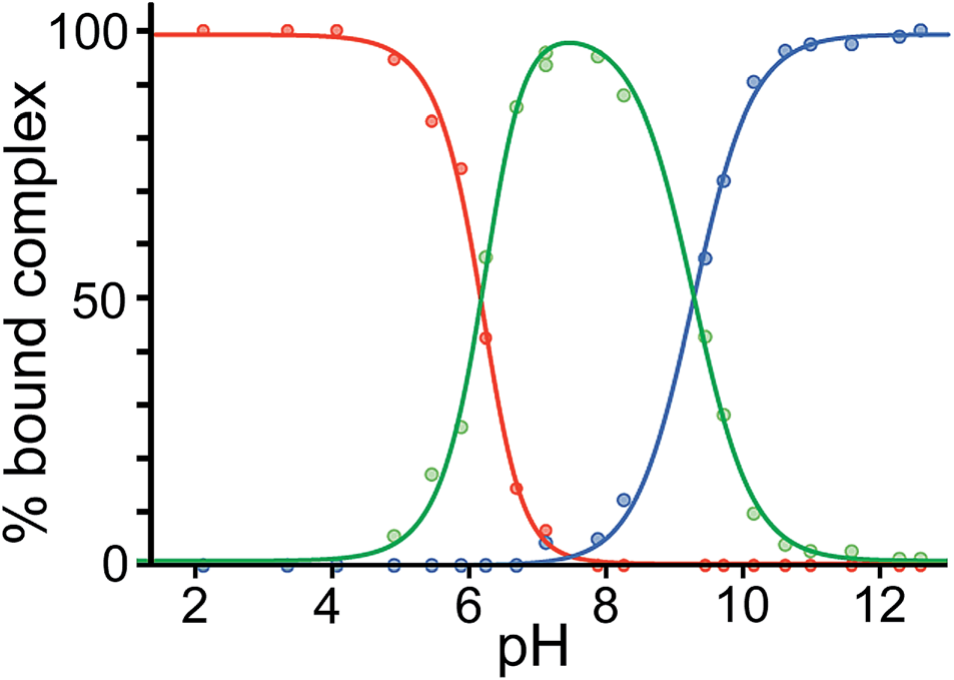
Graphical representation of the data obtained from the NMR spectra in Fig. 5, showing the proportion of each type of complex (red, cage·H_2_**A**; green, cage·**C**; blue, cage·**B**), as a percentage of total complexed cage present, across the pH range. Dots represent measured data. The blue and red lines are calculated fits for pH-dependent binding of monobasic (**B**) and dibasic (H_2_**A**) guests, respectively (see ref. 14); the green line represents the calculated residual fraction of bound guest whose binding is not pH-dependent, *i.e*. cage·**C**.

Finally, we performed a combined experiment to demonstrate switching between all three bound guest states as a function of pH. This is a simple combination of the previous two experiments: a D_2_O solution containing 0.2 mM cage, H_2_**A** (0.75 mM), **B** (7.1 mM) and **C** (0.2 mM) was prepared, and ^1^H NMR spectra were measured over the pH range 3–12. The results are summarized in Fig. 5 and 6. The evolution of ^1^H NMR spectra in the –6 to –11 ppm range (Fig. 5) shows very clearly how, as pH increases, cage·H_2_**A** (dominant complex at low pH) is successively replaced by cage·**C** (dominant complex at neutral pH) and then by cage·**B** (dominant complex at high pH), associated with (i) deprotonation of H_2_**A** to **A**^2–^ at pH ≈ 5 and then (ii) deprotonation of H**B**^+^ to **B** at pH ≈ 11. The proportions of each complex throughout the pH range, expressed as a fraction of total complex concentration, are summarized in Fig. 6 and illustrate very clearly the switching between the three different bound states as a function of pH. The p*K*_a_ values for H_2_**A** and H**B^+^** are far enough apart to allow for near-quantitative conversion between the three bound states: at the extremes, the complexes cage·H_2_**A** (low pH) and cage·**B** (high pH) constitute close to 100% of the total complex present, and at pH 7.5, the population of bound cage is >97% cage·**C** with <2% of each of the other two complexes.

## Conclusions

In conclusion, we have demonstrated how a host cage can select one of three possible guests from a mixture using a single external stimulus (a pH change) – an unprecedented degree of control over guest binding. For any potential applications of molecular containers in which stimulus-responsive guest binding is an important factor, this ability to switch reversibly between any one of *multiple* bound states using a *single* stimulus represents a new level of sophistication and control in host guest chemistry which will expand the range of functions that can be developed.
